# Biphasic Expression of Atypical Chemokine Receptor (ACKR) 2 and ACKR4 in Colorectal Neoplasms in Association with Histopathological Findings

**DOI:** 10.3390/biom11010008

**Published:** 2020-12-23

**Authors:** Paulina Lewandowska, Jaroslaw Wierzbicki, Marek Zawadzki, Anil Agrawal, Małgorzata Krzystek-Korpacka

**Affiliations:** 1 Department of Medical Biochemistry, Wroclaw Medical University, 50-368 Wroclaw, Poland; paulina.lewandowska@student.umed.wroc.pl; 2 Department of Minimally Invasive Surgery and Proctology, Wroclaw Medical University, 50-556 Wroclaw, Poland; jaroslaw.wierzbicki@umed.wroc.pl; 3 Department of Oncological Surgery, Regional Specialist Hospital, 51-124 Wroclaw, Poland; zawadzki@wssk.wroc.pl; 4 Department of Physiotherapy, Wroclaw Medical University, 51-618 Wroclaw, Poland; 5 The 2nd Department of General and Oncological Surgery, Wroclaw Medical University, 50-556 Wroclaw, Poland; anil.agrawal@umed.wroc.pl

**Keywords:** resolution of inflammation, chemoprevention, decoy receptors, colorectal adenomas, colorectal cancer, CC chemokines

## Abstract

Facilitating resolution of inflammation using atypical chemokine receptors (ACKR) as an anticancer strategy is considered but requires a deeper understanding of receptor role in carcinogenesis. We aimed at transcriptional analysis (RTqPCR) of *ACKR2* and *ACKR4* expression in colorectal adenoma-adenocarcinoma sequence in paired normal-neoplastic tissues from 96 polyps and 51 cancers. On average, *ACKR2* was downregulated in neoplastic as compared to non-affected tissue in polyp (by 2.7-fold) and cancer (by 3.1-fold) patients. The maximal downregulation (by 8.2-fold) was observed in adenomas with the highest potential for malignancy and was gradually lessening through cancer stages I-IV, owing to increased receptor expression in tumors. On average, *ACKR4* was significantly downregulated solely in adenocarcinomas (by 1.5-fold), less so in patients with lymph node metastasis, owing to a gradual decrease in *ACKR4* expression among N0-N1-N2 cancers in non-affected tissue without changes in tumors. In adenomas, *ACKR4* downregulation in neoplastic tissue increased with increasing potential for malignancy and contribution of villous growth pattern. *ACKR4* expression increased in non-affected tissue with a concomitant decrease in pathological mucosa. In conclusion, the changes in ACKRs expression occur already in precancerous colorectal lesions, culminating in the adenomas with the highest potential for malignancy. Therefore, chemoprevention by manipulating ACKRs’ expression is worth exploration.

## 1. Introduction

Colorectal cancer (CRC) remains one of the commonest malignancies worldwide [1], ranked second as a cause of cancer deaths in the United States [2]. Surgical resection is a mainstay in CRC treatment, while systemic chemotherapy continues to be the only option for patients with gross metastatic disease. Emerging targeted biological and immune therapies are believed to maximize antitumor effects and minimize toxicity and risk of treatment failure. As such, they are intensively investigated, and novel potential molecular targets for antineoplastic therapy are looked for [3,4].

It is now well accepted that there is a functional link between inflammation and cancer at various stages of its development. Cancer risk is higher in infected patients and patients with chronic inflammatory conditions, and overexpression of inflammatory cytokines and chemokines induce cancer in experimental models [5]. Moreover, targeting inflammation has been shown to reduce the incidence of neoplasms in both animals and humans [5,6,7,8], rendering mediators of inflammation the suitable targets for chemoprevention. Inflammatory cytokines and chemokines also facilitate tumor growth and progression by promoting angiogenesis, invasion, and metastasis [9,10]. Accordingly, Hanahan and Weinberg [11] included the ability to induce and sustain inflammation as the characteristics enabling tumors to acquire their proliferative, antiapoptotic, angiogenic and metastatic potential.

Regarding CRC, the risk in patients with inflammatory bowel disease (IBD) is several times higher than in the general population [5]. However, IBD-related cancer accounts for 1–2% of CRCs, while sporadic cancer, developing through adenoma-adenocarcinoma sequence, occurs in the vast majority of cases. Although considered a less likely trigger of neoplastic transformation, recently gathered evidence indicates a role for inflammation in colorectal adenomas as well. Inflammation is one of the key means by which gut microbiota contribute to the formation and subsequent progression of colorectal adenomas [12]. Correspondingly, immunohistochemical data show infiltration of precancerous lesions with immune cells, with the degree of infiltration being proportional to the adenoma size and grade of dysplasia [13].

Appreciating the supportive role of inflammation, a novel approach for cancer treatment and prevention based on inflammation resolution is being considered. While it is centered mainly on pro-resolving mediators such as resolvins, lipoxins, maresins, and protectins [14], the potential of atypical chemokine receptors (ACKRs), also involved in the resolution of inflammation, is considered [15]. Like chemokines themselves, ACKRs are involved in all steps of cancer development–from its initiation to dissemination [10,15]. The ACKRs scavenge chemokines facilitating their degradation and thus limiting chemokine availability for leukocytes. Moreover, they have been shown to modulate the expression and signaling of canonical chemokine receptors [16]. The ACKRs are expressed mostly on non-leukocyte cells in tumor microenvironment and in cells constituting natural barriers such as gut epithelial cells [15,16]. A family member efficiently depleting most of the inflammatory CC chemokines, ACKR2 (D6/CCBP2), has been shown to be overexpressed in colon samples from IBD patients and in patients with colitis-associated cancer [17] but downregulated in sporadic CRC [18]. Functionally, ACKR2 played a significant role in gut inflammation and cancer as knock-out mice had more severe inflammation and were prone to inflammation-induced cancer [17], gaining the *ACKR2* gene a tumor suppressor label [16]. The ACKR4 (CCRL1/CCX-CKR) is a counterpart for ACKR2, involved in scavenging homeostatic chemokines such as CCL19, CCL21, and CCL25 [15]. Tumor samples from CRC patients have been shown to display lower immunopositivity rates than nontransformed tissue, and ACKR4 protein expression was positively correlated with patients’ survival [19], while transcriptomic analysis of a small set of clinical samples did not show significant differences [18]. In light of growing interest in inflammation resolution as a potential antineoplastic strategy and scarcity of data concerning ACKR expression, the aim of the present study was the analysis of transcriptional patterns of *ACKR2* and *ACKR4* in adenoma-carcinoma sequence in the colorectum in reference to pathological findings and expression of receptors’ ligands.

## 2. Materials and Methods

### 2.1. Study Population

Biobanked bowel samples from 147 individuals were analyzed in the present study, including paired samples (normal and pathological) from 96 patients with colorectal polyps and from 51 patients with colorectal adenocarcinomas, admitted to the Dept. of Minimally Invasive Surgery and Proctology of Wroclaw Medical University for polypectomy or Dept. of Oncological Surgery of Regional Specialist Hospital for curative tumor resection, respectively. Samples were collected prior to any treatment. Cancers were staged pathologically using the TNM grading system (7th edition). The detailed characteristics of patients with colorectal polyps and adenocarcinomas are given in Table 1 and Table 2.

### 2.2. Ethical Approval

The study protocol was approved by the Medical Ethics Committees of Regional Specialist Hospital (#KB/nr 1/rok 2012 from 26 June 2012) and by the Medical Ethics Committees of Wroclaw Medical University (#KB-247/2018 from 24 April 2018). The study was conducted in accordance with the Helsinki Declaration of 1975, as revised in 1983, and informed consent was obtained from all patients.

### 2.3. Analytical Methods

Tissue samples (≤40 mg) soaked and stored in RNAlater (Ambion Inc., Austin TX, USA) at −80 °C were homogenized in lysis buffer with β-mercaptoethanol (Sigma-Aldrich, St. Louis, MO, USA) using ceramic spheres and Fastprep 24 homogenizer (MP Biomedical, Solon, OH, USA). Phenol-chloroform extraction was used to isolate RNA, subsequently purified using PureLink™ RNA Mini Kit (Invitrogen, Thermo Fisher Scientific, Carlsbad, CA, USA) with genomic DNA removal by on-column treatment with DNase (PureLink™ DNase Set; Invitrogen). RNA concentration was quantified, and its purity, as well as integrity, were tested using NanoDrop 2000 spectrophotometer (Thermo Fisher Scientific) and LabChip microfluidic technology on Experion platform with Experion RNA StdSens analysis kits (Bio-Rad, Herkules CA, USA).

Aliquots containing 1000 ng of RNA were reversely transcribed using iScript™ cDNA synthesis kit (Bio-Rad) in C1000 thermocycler (Bio-Rad).

Quantitative (real-time) PCR (qPCR) reactions were conducted in CFX96 real-rime PCR system (Bio-Rad) using SsoFast EvaGreen^®^ Supermix (Bio-Rad) and the following cycling conditions: 30 s activation at 95 °C, 5 s denaturation at 95 °C, annealing/extension for 5 s at 61 °C, 40 cycles, followed by melting step (60–95 °C with fluorescent reading every 0.5 °C). The reaction mixture contained 10 μL of 2× SsoFast EvaGreen^®^ Supermix, 2 μL of cDNA (diluted 1:5), 1 μL of each 10 nM forward and reverse target-specific primers and water up to 20 μL. Primers, using intron-spanning sequences proposed by OriGene (www.origene.com) as follows: 5′-gactacgcactccaggtaacag-3′ (*ACKR2* forward), 5′-aagccttcaggtactggcggaa -3′ (*ACKR2* reverse), 5′-gtctctggaatgcagtttctggc-3′ (*ACKR4* forward), and 5′-ggtatgctcagcaagatggcag-3′ (*ACKR4* reverse) were synthesized by Genomed (Warsaw, Poland).

The obtained Cq values of technical replicates were averaged. For each analyzed gene, the geometric mean of all Cq values was subtracted from sample Cq, yielding ΔCq. The ΔCq values were then linearized by 2^ΔCq conversion and normalized to internal control. The geometric mean of *PPIA* (5′-ggcaaatgctggacccaacaca-3′ forward and 5′-tgctggtcttgccattcctgga-3′ reverse) and *RPLP0* (5′-tcacaacaagcataccaagaagc-3′ forward and 5′-gtatccgatgtccacaatgtcaag-3′ reverse) expression served as a reference in the current study. The obtained values are referred to as a normalized relative quantity (NRQ) [20] and subjected to statistical analysis. Reference gene selection was based on our earlier study, demonstrating that *PPIA* and *RPLP0* are the most suitable pair of genes for studies on bowel tissues from CRC patients [21].

### 2.4. Statistical Analysis

Data were tested for normality of distribution (Kolmogorov–Smirnov test) and homogeneity of variances (Levene test). Log-transformation was used to obtain the normality of distribution and/or to improve the homogeneity of variances. A paired analysis was conducted using a *t*-test for paired samples and unpaired analysis-using a *t*-test (two-group analysis) or one-way analysis of variance (ANOVA; multigroup comparisons) in case of normal distribution and homogeneity of variances and Kruskal–Wallis H test in case of non-normal distribution and/or nonhomogenous variances. The following post hoc tests were applied: Students–Newman–Keuls for ANOVA and Conover for Kruskal–Wallis. Correlation analysis was conducted using Spearman rank correlation (ρ) or Pearson’s correlation (r). The following descriptors were used for interpretation of correlation results: < 0.1 as negligible; 0.1–0.39 as weak; 0.4–0.69 as moderate; 0.7–0.89 as strong; 0.9–1.0 as very strong [22]. All tests were two-sided, and p values < 0.05 were considered statistically significant. Statistical analysis was conducted using MedCalc^®^ Statistical Software version 19.5.3 (MedCalc Software Ltd., Ostend, Belgium; https://www.medcalc.org; 2020).

## 3. Results

### 3.1. ACKR2 and ACKR4 in Colorectal Adenomas

**Figure 1:**
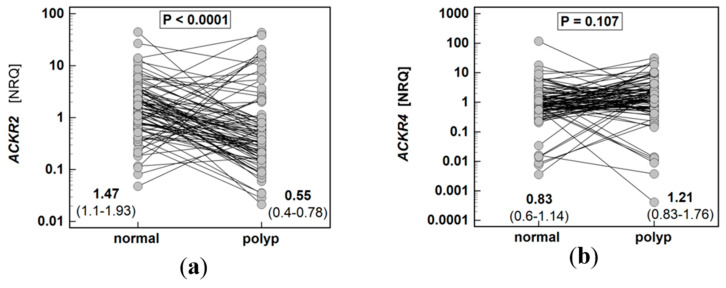
Expression of atypical chemokine receptors (ACKR) in colorectal polyps: (**a**) ACKR2; (**b**) ACKR4. Data presented as dot-plots and mean (95% confidence interval) and analyzed using *t*-test for paired samples. NRQ, normalized relative quantity.

The expression of *ACKR2* in polyps was 2.7-fold lower than in patient-matched macroscopically normal tissues, while *ACKR4* did not differ significantly (Figure 1).

#### 3.1.1. Association with Histological Type

Expression rate (normal-to-adenoma) of *ACKR2* was insignificantly higher in villous adenomas, which was a result of insignificantly lower receptor expression in polyp tissue of villous as compared to tubular and tubulovillous type (Supplementary Materials Figure S1).

**Figure 2:**
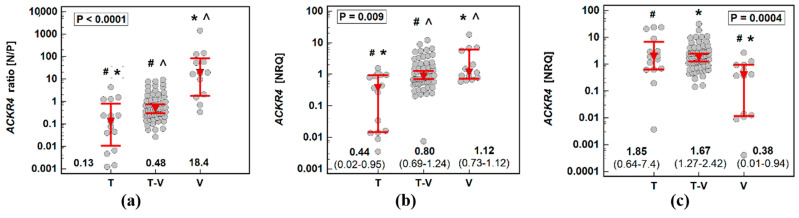
Impact of adenoma histological type on *ACKR4* expression: (**a**) normal–to-polyp expression ratio (N/P); (**b**) in normal tissue; (**c**) in adenomas. Data presented as dot-plots with medians with 95% confidence interval (figures below the dot-plots and red triangles with whiskers) and analyzed using a Kruskal–Wallis H test with a Conover post hoc test. Groups differing significantly in a post hoc analysis are indicated by the same type of symbol: *, #, or ^. T, tubular adenomas; T-V, tubulovillous adenomas; V, villous adenomas; NRQ, normalized relative quantity.

The difference between *ACKR4* expression in normal tissue and adenoma was significantly dependent on histological type of adenoma, shifting from receptor overexpression in adenomas as compared to normal tissue in patients with tubular adenomas to its overexpression in normal tissue as compared to adenomas in patients with villous adenomas (Figure 2a). This resulted from a concomitant change in expression pattern in normal tissue and adenomas with significantly lower *ACKR4* expression in normal tissue from patients with tubular adenomas (Figure 2b) and significantly lower *ACKR4* expression in adenomas from patients with the villous type (Figure 2c).

**Figure 3:**
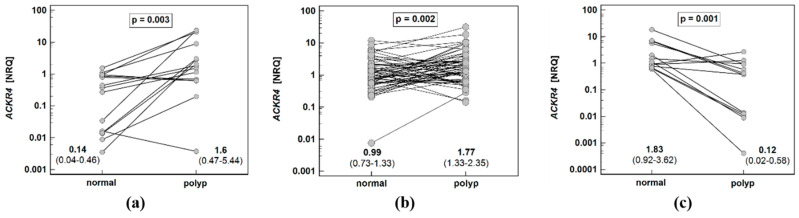
Pairwise analysis of ACKR4 expression in patients stratified by adenoma histological type: (**a**) tubular adenomas; (**b**) tubulovillous adenomas; (**c**) villous adenomas. Data presented as dot-plots and mean (95% confidence interval) and analyzed using *t*-test for paired samples. NRQ, normalized relative quantity.

Considering the dependence of ACKR4 expression on histological type, we repeated pairwise analysis separately for patients with adenomas of tubular, tubule-villous, and villous growth patterns. Even though *ACKR4* expression in normal mucosa as compared to corresponding patient-matched polyps was not significantly different on average (Figure 1b), it was significantly lower in tubular adenomas (by 11.4-fold) and tubulovillous adenomas (by 1.8-fold) and significantly higher (by 15.8-fold) in a subgroup of patients with villous adenomas (Figure 3).

#### 3.1.2. Association with Dysplasia Grade

Neither *ACKR2* nor *ACKR4* expression ratios (normal-to-polyp) differed significantly depending on dysplasia grade (Supplementary Materials Figure S2).

#### 3.1.3. Association with Adenoma Size and Location

Adenoma size (Supplementary Materials Figure S3) or its location in the colorectum (Supplementary Materials Figure S4) had no significant effect on *ACKR2* or *ACKR4* expression.

#### 3.1.4. Association with Cumulated Potential for Malignancy

Hyperplastic polyps of the serrated pathway—as well as small adenomas with low-grade dysplasia and with solely tubular growth pattern—are considered to have a small potential for malignancy while adenoma size ≥10 mm, high-grade hyperplasia and presence of villous growth pattern are each considered risk factors for malignancy [23,24]. Therefore, we stratified patients with polyps based on the number of factors increasing the risk of malignancy. Score one was assigned to patients with none or one of these risk factors, score two to patients with two risk factors, and score three was assigned to patients having three high-risk factors.

**Figure 4:**
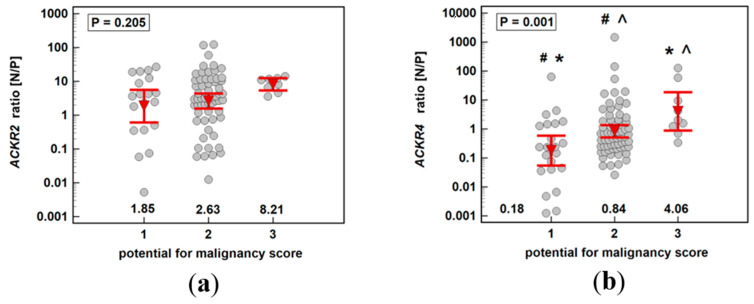
Impact of polyp cumulated risk for malignancy on expression ratio (normal-to-polyp) (N/P) of: (**a**) *ACKR2*; (**b**) *ACKR4*. Data presented as means with 95% confidence interval (figures below the dot-plots and red triangles with whiskers) and analyzed using one-way analysis of variance with Students–Newman–Keuls post hoc test. Groups differing significantly in a post hoc analysis are indicated by the same type of symbol: *, #, or ^.

Expression ratios (normal-to-polyp) of *ACKR2* for patients with polyps with all three risk factors were insignificantly higher than in patients with no or few risk factors. Regardless of the potential for malignancy score, *ACKR2* was downregulated in polyps as compared to corresponding normal mucosa as indicated by the fact that normal-to-polyp expression ratios exceeded one in all cases (Figure 4a). The expression rates of *ACKR4* differed significantly between patients with polyps at various risk for malignancy. Interestingly, only in patients at the highest risk, the receptor was downregulated in polyp as compared to corresponding normal mucosa (normal-to-polyp expression ratio exceeding four). As indicated by normal-to-polyp expression ratios below one in the remaining cases, *ACKR4* expression was lower in normal mucosa than patient-matched polyps in patients at lower risk for malignancy. In fact, in a group with none or one risk factor (malignancy score one), *ACKR4* expression was upregulated by 5.6-fold in polyps as compared to corresponding normal mucosa (P/N = 5.6 as a reciprocal of N/P = 0.18 from Figure 4b).

### 3.2. ACKR2 and ACKR4 in Colorectal Adenocarcinomas

**Figure 5:**
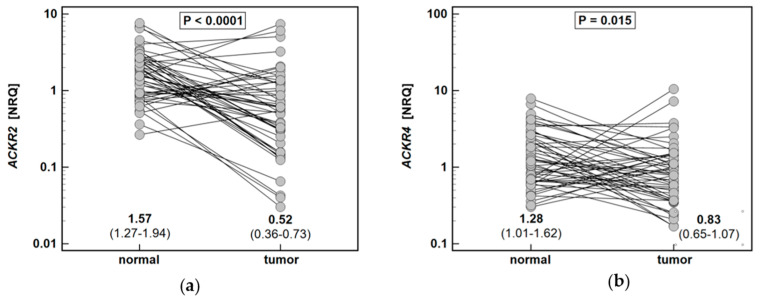
Expression of atypical chemokine receptors (ACKR) in colorectal adenocarcinomas: (**a**) *ACKR2*; (**b**) *ACKR4*. Data presented as dot-plots and mean (95% confidence interval) and analyzed using *t*-test for paired samples. NRQ, normalized relative quantity.

The expression of *ACKR2* and *ACKR4* in the tumor was, respectively, 3.1-fold and 1.5-fold lower than in patient-matched macroscopically normal tissue (Figure 5).

#### 3.2.1. Association with Depth of Tumor Invasion

**Figure 6:**
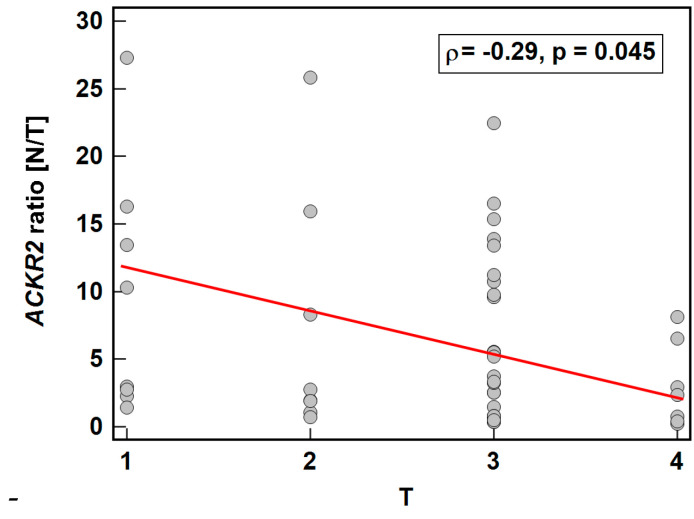
Association between *ACKR2* expression and depth of tumor invasion. Data presented as Spearman correlation coefficient rho (ρ) represented graphically as a red trend line. N/T, expression ratio normal-to-tumor.

As depicted in Figure 6, the expression ratio (normal-to-tumor) of *ACKR2* decreased along with an increasing depth of tumor invasion (T0/1-T2-T3-T4), while that of *ACKR4* did not display significant association (ρ = −0.15, *p* = 0.283).

#### 3.2.2. Association with Lymph Node Metastasis

*ACKR2* did not show any significant differences in expression ratio (*p* = 0.534) or expression in normal tissue (*p* = 0.919) or in tumors (*p* = 0.563) with respect to lymph node metastasis.

**Figure 7:**
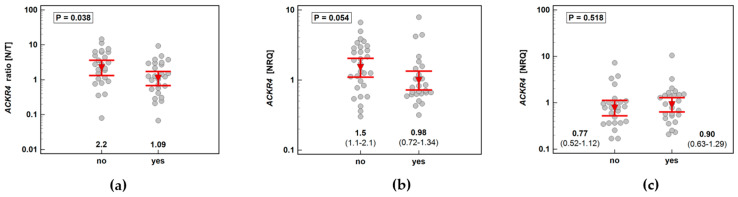
Impact of lymph node involvement on *ACKR4* expression: (**a**) normal–to-tumor expression ratio (N/T); (**b**) in macroscopically normal tissue; (**c**) in tumors. Data presented as dot-plots with means with 95% confidence interval (figures below the dot-plots and red triangles with whiskers) and analyzed using *t*-test for independent samples. NRQ, normalized relative quantity.

**Figure 8:**
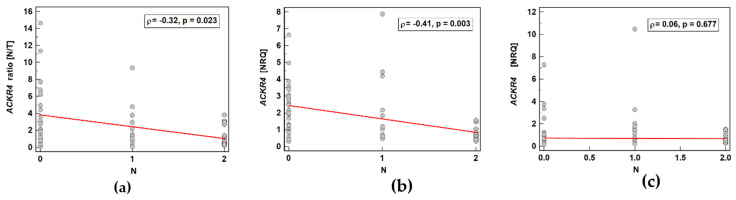
Correlation between cancer N stage and *ACKR4* expression: (**a**) as a normal–to-tumor expression ratio (N/T); (**b**) in macroscopically normal tissue; (**c**) in tumors. Data presented as Spearman correlation coefficient rho (ρ) represented graphically as a red trend line. NRQ, normalized relative quantity.

*ACKR4* was overexpressed in macroscopically normal tumor-adjacent tissue as compared to matched tumors solely in patients without lymph node metastasis, which reflected higher receptor expression in normal tissue in N0 patients with lack of differences between normal and tumor tissue in patients with and without metastasis (Figure 7). As depicted in Figure 8, the ACKR4 expression ratio (normal-to-tumor) gradually decreased along with increasing lymph node involvement (N0-N1-N2). It reflected the gradual decrease in ACKR4 expression in normal tissue, but not in tumors (Figure 8).

#### 3.2.3. Association with Tumor Grade

The expression ratio (normal-to-tumor) of *ACKR2* insignificantly decreased along with increasing tumor grade (G1-G2-G3) (ρ = −0.27, *p* = 0.069), owing to insignificant increase of *ACKR2* expression in tumors (ρ = 0.27, *p* = 0.065).

Likewise, the expression ratio of *ACKR4* insignificantly decreased (ρ = −0.28, *p* = 0.055), but owing to insignificant decrease of *ACKR4* expression in normal tissue (ρ = −0.27, *p* = 0.068).

#### 3.2.4. Association with Tumor Location 

Tumor location in the colorectum had no significant effect on expression ratios of *ACKR2* or *ACKR4* (Supplementary Materials Figure S5).

### 3.3. ACKR Expression and Malignant Potential Through Adenoma-adenocarcinoma Sequence

To discern the expression patterns of ACKRs through adenoma-adenocarcinoma transition to the receptor association with adenoma potential for malignancy, we added cancer stage in adenocarcinoma patients. Therefore, in addition to scores from one to three assigned to patients with polyps, CRC patients were assigned scores from four to seven, which corresponded with the disease stages I-IV.

**Figure 9:**
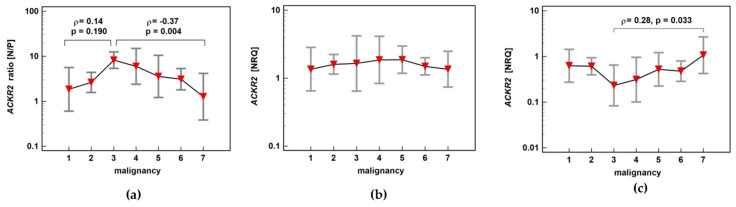
Impact of polyp risk for malignancy and cancer malignant potential on (**a**) *ACKR2* expression ratio (normal-to-pathological) (N/P); (**b**) *ACKR2* expression in normal mucosa; (**c**) *ACKR2* expression in pathological tissue (adenoma or adenocarcinoma). Data presented as means with 95% confidence interval (red triangles with whiskers). The relationship was analyzed using Spearman rank correlation and expressed as correlation coefficient *ρ*. NRQ, normalized relative quantity.

*ACKR2* expression ratio (normal-to-pathological) increased insignificantly up-to score three and subsequently decreased significantly along with increasing malignancy, indicating maximal receptor downregulation in adenomas with the highest potential for malignancy. The individual analysis of expression level in normal and pathological mucosa showed stable expression in macroscopically normal tissue and an insignificant drop at score three with the subsequent significant gradual increase in *ACKR2* expression through increasing stages of cancer advancement (Figure 9).

**Figure 10:**
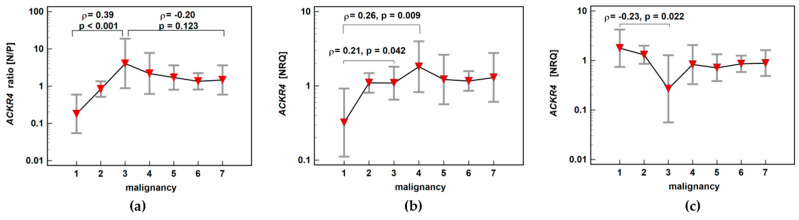
Impact of polyp risk for malignancy and cancer malignant potential on (**a**) *ACKR4* expression ratio (normal-to-pathological) (N/P); (**b**) *ACKR4* expression in normal mucosa; (**c**) *ACKR4* expression in pathological tissue (adenoma or adenocarcinoma). Data presented as means with 95% confidence interval (red triangles with whiskers). The relationship was analyzed using Spearman rank correlation and expressed as correlation coefficient *ρ*. NRQ, normalized relative quantity.

*ACKR4* expression ratio (normal-to-pathological) increased significantly up-to score three and subsequently decreased insignificantly along with increasing malignancy, also indicating maximal receptor downregulation in adenomas with the highest potential for malignancy. The correlation between adenoma potential for malignancy and relative receptor downregulation resulted from concomitant changes in normal and neoplastic tissue. In non-affected mucosa, *ACKR4* expression increased with a peak at score 4, that is, in patients with stage I CRC. In neoplastic tissue, *ACKR4* expression decreased gradually with a minimum of expression at score 3, that is, in patients with adenomas of the highest potential for malignancy (Figure 10).

### 3.4. Correlation Patterns with ACKR Ligands

We examined the correlation of ACKR2 with its ligands, that is, chemokines CCL2, CCL3, CCL4, CCL7, and CCL8. In adenocarcinomas, the expression ratios (normal-to-pathological) of ACKR2 were positively correlated with all those chemokines while in polyps, with CCL3 and less markedly with CCL4 and CCL7 (Table 3). Scatterplots of significant correlations are presented in Supplementary Materials (Figure S6).

There was a weak correlation between expression ratios of ACKR4 and its ligand CCL19 in polyps (r = 0.26, *p* = 0.014) resulting from a slightly stronger correlation between ACKR4 and CCL19 expression in pathological tissue (r = 0.33, *p* = 0.001). In adenocarcinomas, there was no correlation between ACKR4 and CCL19 expression ratios (r = 0.04, *p* = 0.764), although ACKR4 and CCL19 expressions in tumors were positively correlated (r = 0.29, *p* = 0.041).

## 4. Discussion

To the best of our knowledge, this is the first report analyzing *ACKR2* and *ACK4* expression in colorectal adenoma-adenocarcinoma sequence. We showed a biphasic pattern with the most pronounced receptors’ downregulation in neoplastic tissue as compared to normal mucosa in adenomas with the highest potential for malignancy. As indicated by a recent review of Sjoberg et al. [15], there are only a few reports demonstrating ACKR2 and ACKR4 association with CRC, but none regarding adenomas. ACKR2 was investigated in the context of bowel inflammation and colitis-associated cancer (CAC) by Vetrano et al. [17]. The authors have shown colonic lymphatic vessels and leukocytes to be immunopositive for ACKR2. The immunoreactivity of lymphatic vessels, but not leukocytes, was significantly higher in samples derived from patients with active IBD or CAC than those of normal mucosa obtained from patients with CRC, polyps or diverticulosis. Results to the contrary were reported by Langenes et al. [18], who demonstrated median 15-fold downregulation of *ACKR2* mRNA in colonic tumors. Likewise, we showed that *ACKR2* expression in adenomas and sporadic CRC was downregulated in pathological tissue as compared to adjacent macroscopically normal mucosa. The immunoreactivity and mRNA expression data for ACKR2 are claimed to be mainly consistent [25]. Therefore, the discrepancy between studies is likely to be associated with dissimilarities in molecular pathways leading to the neoplastic transformation between CAC and sporadic CRC rather than being a manifestation of differences between receptor expression at protein and mRNA level. Receptor downregulation observed in our and Langenes et al.’s [18] cohorts are consistent with a tumor suppressor role [16] and dominantly tumor-inhibitory effects [15] attributed to ACKR2. It is also corroborated by immunohistochemical (IHC) findings in cervical cancer [26], although data regarding breast [27] and gastric cancer [28] have shown only similar tendencies. Gene expression analysis conducted here demonstrated that the degree of *ACKR2* downregulation was similar in adenomas and adenocarcinomas. However, the pattern of association with neoplasms advancement, that is, the potential for malignancy in adenomas and TNM stage in CRC, was different. The downregulation of *ACKR2* expression in adenomas insignificantly increased along with cumulated risk for malignancy, expressed as a sum of high-risk factors such as the presence of villous component, high-grade of dysplasia, and adenoma size exceeding 10 mm [24]. The highest normal-to-pathological expression rates, and thus the most pronounced *ACKR2* downregulation in neoplastic tissue, were demonstrated in polyps with the highest potential for malignancy, that is, in large adenomas with a high-grade of dysplasia and villous growth pattern. As compared to the 2.7-fold lower expression on average in neoplastic tissue, patients with score three adenomas had *ACKR2* expression lower by 8.2-fold. In turn, the actual adenocarcinomas had less pronounced *ACKR2* downregulation. Moreover, the downregulation significantly decreased along with the disease advancement, although even at stage IV CRC, *ACKR2* remained downregulated in the tumor as compared to matched normal mucosa (by 1.3-fold). Both in adenomas and adenocarcinomas, the observed *ACKR2* downregulation resulted from changes in expression in neoplastic tissue while receptor expression in normal mucosa remained relatively stable at subsequent steps of adenoma-adenocarcinoma sequence. Of the individual TNM staging system components, *ACKR2* expression was negatively correlated with the depth of tumor invasion (T). This observation contradicts findings from Langenes et al.’s study [18], in which T3/T4 tumors had more marked downregulation than T1/T2 tumors. As both studies had a rather limited number of T1 and T2 cases and the correlation observed here was rather weak, of borderline significance, and not supported by the significant association in a separate analysis of normal and tumor tissues, the issue needs to be addressed in a larger study. The scarce data regarding other cancers have indicated a negative association of ACKR2 protein expression in tumors with TNM stage in breast [27] and gastric cancer [28], lymph node metastasis in breast cancer [27,29], tumor size in cervical cancer [26], histological grade in gastric cancer [28], and with the recurrence of cervical cancer [26].

A similar biphasic expression pattern through the adenoma–-adenocarcinoma sequence was displayed by *ACKR4*. Maximal receptor downregulation was also associated with adenomas of the highest potential for malignancy, with 4.1-fold downregulation in neoplastic tissue. Downregulated ACKR4 protein expression in the tumor as compared to normal tissue has been reported in cervical [26] and liver cancer [30] as well as in nasopharyngeal carcinoma [31], but not in gastric [28] or breast cancers [27]. Contrary to *ACKR2*, the *ACKR4* was not always downregulated in neoplastic mucosa as compared to normal tissue. In fact, *ACKR4* in polyps of none and low malignant potential was upregulated (by 5.6-fold). Likewise, adenomas with tubular and tubulovillous growth patterns overexpressed *ACKR4* and receptor downregulation in adenomas as compared to patient-matched normal tissue was observed solely in patients with dominant villous growth patterns. The descending tendency of *ACKR4* expression ratio along with increasing cancer stage in adenocarcinomas was not statistically significant. However, the receptor expression was significantly dependent on lymph node involvement. The *ACKR4* was downregulated in tumors solely in patients without lymph node metastasis (by 2.2-fold), and its expression rates were inversely related to an increasing N stage. Interestingly, not the changes in *ACKR4* expression in tumors, but those in corresponding normal tissue accounted for the observed inverse relationship. Regarding the link between receptor expression and overall malignant potential, the changes were occurring in both normal and neoplastic tissue. Of note, it has been repeatedly demonstrated that the macroscopically normal tissue adjacent to tumors might be already altered at the molecular level, preceding histological and morphological changes, in a manner reflecting disease advancement [32,33,34]. This phenomenon—referred to as the “tumor molecular margin”—is clinically important as it is held responsible for cancer recurrence following surgery and for the simultaneous occurrence of multiple tumors [35,36,37].

Previously ACKR4 in CRC has been examined at the protein level by Zhu et al. [19] and at mRNA level, although on a small set of samples (n = 13), by Langenes et al. [18]. Corroborating our observations, Zhu et al. [19] reported a higher ACKR4-positive protein expression rate in normal mucosa than tumors. However, IHC findings on the association of ACKR4 protein expression with pathological findings have shown decreasing receptor immunopositivity along with increasing CRC stage and its lower expression in patients with lymph node metastasis [19]. Consistently, functional in vitro studies have shown that receptor overexpression in colonic cancer cell lines had no effect on cell proliferation but inhibited their migratory and invasive properties. Mechanistically, enhanced ACKR4 expression negatively affected the expression of CCR7, CCR9, CXCR5, and CXCR4 - functional receptors for ACKR4′s ligands [19]. Furthermore, in an animal model of nasopharyngeal carcinoma, the loss of receptor promoted lymph node metastasis and tumor growth. Mechanistically, loss of ACKR4 was associated with CCL21 accumulation and resulting in the increased proliferation rate of the nasopharyngeal carcinoma cell line, induction of genes involved in epithelial-mesenchymal transition, and upregulation of matrix metalloproteinases 2 and 9 [31]. In line with Zhu et al. [19] observations regarding CRC, the negative association of ACKR4 expression with cancer stage [26,27,28], lymph node metastasis [26,27,38], and dedifferentiation [30] has been repeatedly noted in clinical samples from other cancer types as well. Unlike IHC studies on ACKR4 protein, which are conducted solely on pathological tissue, paired analysis of gene expression showed the *ACKR4* downregulation to be less marked in more dedifferentiated tumors and in patients with lymph node metastasis. Detailed analysis showed that mRNA level in pathological tissue is relatively unaltered. There is indeed the “the more advanced/aggressive tumor, the lower ACKR4 expression” trend, but as it concerns normal tissue, the normal-to-expression rate shows apparently less downregulated ACKR4 in more advanced/aggressive cancers.

Although non-significantly associated with a cumulative potential for malignancy, the expression of *ACKR4* in adenomas was significantly dependent on its growth pattern. Normal-to-polyp expression rates were the lowest in tubular adenomas and increased with increasing contribution of villous growth pattern. Detailed analysis showed that the observed effect was the result of the concomitant change in normal and neoplastic tissue. While patients with tubular adenomas had significantly lower *ACKR4* expression than those with tubulovillous and villous adenomas regarding normal tissue, the lowest receptor expression in neoplastic tissue was observed in adenomas with prevalent villous growth pattern.

Neoplasm location may affect tumor biological behavior, effectiveness of treatment, and ultimately patient’s prognosis [39]. Subsite heterogeneity of tumors arises from distinct genetic alterations [40,41] and differences in gene and protein expression patterns between various sublocations in the colorectum [42]. Moreover, subsite heterogeneity is also reflected at the systemic level [43], including ACKR2 ligands MIP-1α (CCL3) and MIP-1β (CCL4) [44]. Indeed, Langenes et al. [18] hinted at more pronounced downregulation of *ACKR2* mRNA expression in tumors located in the sigmoid colon as compared to the cecum. Therefore, the possible association between ACKRs expression and anatomical subsite was investigated. We found only insignificantly more marked *ACKR2* and *ACKR4* downregulation in right-sided tumors. Still, the observation was consistent with higher systemic concentrations of MIP-1α and MIP−1β [44] and may contribute to their least favorable characteristics. Right-sided colonic cancers are considered to be less differentiated and thus more aggressive, more advanced upon diagnosis and likely to be resistant to chemotherapy, associated with a higher risk for second primary CRC and, ultimately, linked with worse prognosis [45,46,47]

The dominant mechanism by which ACKR receptors are involved in the resolution of inflammation is chemokine scavenging and directing for degradation. In addition, mostly via their effect on chemokines and their receptors, the ACKRs impact cancer development [15]. Correspondingly, Savino et al. [48] demonstrated that ACKR2 downregulation in Kaposi sarcoma cells yielded larger tumors, which was accompanied by the recruitment of monocytes mediated by CCL2. In addition, reduced receptor expression triggered the differentiation of macrophages into pro-angiogenic and pro-tumor phenotype. Others, in turn, have shown that inhibitory effects of ACKR4 overexpression on tumor growth are associated with downregulating the expression of functional chemokine receptors and thwarting their signaling [19,30,38]. As such, we conducted the analysis of coexpression of ACKRs and their ligands in adenomas and adenocarcinomas. Generally, there were positive correlations between ACKRs and their ligands. In patients with polyps, *ACKR2* markedly correlated solely with *CCL3*, which was also the strongest correlation in CRC patients, although in cancer the significant correlations were present between the receptor and all examined chemokines from MIP and MCP family. The positive correlation between *ACKR4* expression in neoplastic tissue and *CCL19* was weak but present in both polyps and adenocarcinomas.

The current study is restricted to transcriptional analysis, which, although fully quantitative and sensitive, does not allow to determine the cellular origin of receptor expression, which should be considered as a limitation. Potential receptor sources in the colon include lymphatic endothelial cells and leukocytes in the case of *ACKR2* and lymphatic endothelium and epithelial cells in the case of *ACKR4* [16]. The material analyzed by RTqPCR is heterogeneous with respect to its cellular composition, and it cannot be excluded that the observed differences in receptor expression reflect, at least to some degree, the altered cellular landscape in neoplasms and their immediate surrounding. Therefore, it would be of interest to involve more sophisticated techniques, such as single-cell RNA sequencing, in future research on ACKRs.

## 5. Conclusions

In the colorectum, the alteration in the expression of atypical chemokine receptors *ACKR2* and *ACKR4* occurs already in premalignant lesions, with the receptor downregulation culminating at the verge of malignant transformation, that is, in large adenomas with high-grade dysplasia and dominant villous growth pattern. As such, restoring their expression as a strategy of colorectal cancer chemoprevention is worth exploration.

## Figures and Tables

**Table 1:** Characteristics of patients with colorectal polyps.

Parameter	Characteristics
Sex distribution (F/M), *n*	43/53
Age (y), mean (95% CI)	65.1 (62.7–67.4)
Histological type, *n*:	
tubular adenoma	15
tubulovillous adenoma	60
villous adenoma	13
hyperplastic polyps	8
Grade of dysplasia, *n*:	
low	71
high	17
Adenoma size, *n*:	
<10 mm	18
10–19 mm	45
≥20 mm	25
Polyp location, *n*:	
left colon	48
right colon	25
rectum	23

*N*, number of observations; F/M, female-to-male ratio; y, years; CI, confidence interval.

**Table 2:** Characteristics of patients with colorectal adenocarcinomas.

Parameter	Characteristics
Sex distribution (F/M), *n*	21/30
Age (y), mean (95% CI)	67.5 (64.5–70.5)
Cancer TNM stage (0/I/II/III/IV), *n*	8/5/11/23/4
Depth of tumor invasion (T0-1/T2/T3/T4), *n*	8/8/27/8
Lymph node metastasis (N0/N1/N2), *n*	25/13/13
Distant metastasis (M0/M1), *n*	47/4
Histological grade (G1/G2/G3), *n*	4/35/8
Primary tumor location, *n*:	
left colon	17
right colon	17
rectum	17

*N*, number of observations; F/M, female-to-male ratio; y, years; CI, confidence interval; TNM, tumor-node-metastasis cancer staging system.

**Table 3:** Correlation pattern of expression ratios (normal-to-pathological) of *ACKR2* and its ligands.

Gene	Polyps	Adenocarcinomas
*CCL2*	r = 0.15, *p* = 0.170	r = 0.44, *p* = 0.002
*CCL3*	r = 0.41, *p* < 0.001	r = 0.54, *p* < 0.0001
*CCL4*	r = 0.25, *p* = 0.022	r = 0.43, *p* = 0.002
*CCL7*	r = 0.27, *p* = 0.017	r = 0.52, *p* = 0.0001
*CCL8*	r = 0.13, *p* = 0.253	r = 0.43, *p* = 0.002

Data presented as Pearson’s correlation coefficients (r).

## Data Availability

Data sharing is not applicable to this article.

## Supplementary Materials

The supplementary materials are available online at https://www.mdpi.com/2218-273X/11/1/8/s1, Figure S1: Impact of adenoma histological type on ACKR2 expression, Figure S2: Impact of dysplasia grade on adenoma expression of *ACKR2* and *ACKR4*, Figure S3: Impact of adenoma size on the expression of *ACKR2* and *ACKR4*, Figure S4: Impact of adenoma location on the expression of *ACKR2* and *ACKR4*, Figure S5: Impact of tumor location on the expression of *ACKR2* and *ACKR4*, Figure S6: Correlation between *ACKR2* and chemokine expression.

## References

[1] Bray F., Ferlay J., Soerjomataram I., Siegel R.L., Torre L.A., Jemal A. (2018). Global cancer statistics 2018: GLOBOCAN estimates of incidence and mortality worldwide for 36 cancers in 185 countries. CA Cancer J. Clin..

[2] Siegel R.L., Miller K.D., Goding Sauer A., Fedewa S.A., Butterly L.F., Anderson J.C., Cercek A., Smith R.A., Jemal A. (2020). Colorectal cancer statistics, 2020. CA Cancer J. Clin..

[3] Noel M.S. (2017). Biologics in bowel cancer. J. Gastrointest Oncol..

[4] Bever K.M., Le D.T. (2017). An expanding role for immunotherapy in colorectal cancer. J. Natl. Compr. Cancer Netw..

[5] Kraus S., Arber N. (2009). Inflammation and colorectal cancer. Curr. Opin. Pharmacol..

[6] Saini M.K., Sanya S.N. (2014). Targeting angiogenic pathway for chemoprevention of experimental colon cancer using C-phycocyanin as cyclooxygenase-2 inhibitor. Biochem. Cell Biol..

[7] Meyskens F.L., McLaren C.E., Pelot D., Fujikawa-Brooks S., Carpenter P.M., Hawk E., Kelloff G., Lawson M.J., Kidao J., McCracken J. (2008). Difluoromethylornithine plus sulindac for the prevention of sporadic colorectal adenomas: A randomized placebo-controlled, double-blind trial. Cancer Prev. Res..

[8] Burke C.A., Dekker E., Samadder N.J., Stoffel E., Cohen A. (2016). Efficacy and safety of eflornithine (CPP-1X)/sulindac combination therapy versus each as monotherapy in patients with familial adenomatous polyposis (FAP): Design and rationale of a randomized, double-blind, Phase III trial. BMC Gastroenterol..

[9] Mukaida N., Sasaki S., Baba T. (2014). Chemokines in cancer development and progression and their potential as targeting molecules for cancer treatment. Mediat. Inflamm..

[10] Massara M., Bonavita O., Mantovani A., Locati M., Bonecchi R. (2016). Atypical chemokine receptors in cancer: Friends or foes?. J. Leukoc. Biol..

[11] Hanahan D., Weinberg R.A. (2011). Hallmarks of cancer: The next generation. Cell.

[12] Vacante M., Ciuni R., Basile F., Biondi A. (2020). Gut Microbiota and Colorectal Cancer Development: A Closer Look to the Adenoma-Carcinoma Sequence. Biomedicines.

[13] McLean M.H., Murray G.I., Stewart K.N., Norrie G., Mayer C., Hold G.L., Thomson J., Fyfe N., Hope M., Mowat N.A. (2011). The Inflammatory Microenvironment in Colorectal Neoplasia. PLoS ONE.

[14] Zhang Q., Zhu B., Li Y. (2017). Resolution of Cancer-Promoting Inflammation: A New Approach for Anticancer Therapy. Front. Immunol..

[15] Sjöberg E., Meyrath M., Chevigné A., Östman A., Augsten M., Szpakowska M. (2020). The diverse and complex roles of atypical chemokine receptors in cancer: From molecular biology to clinical relevance and therapy. Adv. Cancer Res..

[16] Bonecchi R., Graham G.J. (2016). Atypical Chemokine Receptors and Their Roles in the Resolution of the Inflammatory Response. Front. Immunol..

[17] Vetrano S., Borroni E.M., Sarukhan A., Savino B., Bonecchi R., Correale C., Arena V., Fantini M., Roncalli M., Malesci A. (2010). The lymphatic system controls intestinal inflammation and inflammation-associated Colon Cancer through the chemokine decoy receptor D6. Gut.

[18] Langenes V., Svensson H., Börjesson L., Gustavsson B., Bemark M., Sjöling Å., Quiding-Järbrink M. (2013). Expression of the chemokine decoy receptor D6 is decreased in colon adenocarcinomas. Cancer Immunol. Immunother..

[19] Zhu Y., Tang W., Liu Y., Wang G., Liang Z., Cui L. (2014). CCX-CKR expression in colorectal cancer and patient survival. Int. J. Biol. Markers.

[20] Vandesompele J., De Preter K., Pattyn F., Poppe B., Van Roy N., De Paepe A., Speleman F. (2002). Accurate normalization of real-time quantitative RT-PCR data by geometric averaging of multiple internal control genes. Genome Biol..

[21] Krzystek-Korpacka M., Diakowska D., Bania J., Gamian A. (2014). Expression stability of common housekeeping genes is differently affected by bowel inflammation and cancer: Implications for finding suitable normalizers for inflammatory bowel disease studies. Inflamm. Bowel. Dis..

[22] Schober P., Boer C., Schwarte L.A. (2018). Correlation Coefficients: Appropriate Use and Interpretation. Anesth. Analg..

[23] East J.E., Atkin W.S., Bateman A.C., Clark S.K., Dolwani S., Ket S.N., Leedham S.J., Phull P.S., Rutter M.D., Shepherd N.A. (2017). British Society of Gastroenterology position statement on serrated polyps in the colon and rectum. Gut.

[24] Fleming M., Ravula S., Tatishchev S.F., Wang H.L. (2012). Colorectal carcinoma: Pathologic aspects. J. Gastrointest. Oncol..

[25] The Human Protein Atlas. https://www.proteinatlas.org/ENSG00000144648-ACKR2/pathology.

[26] Hou T., Liang D., Xu L., Huang X., Huang Y., Zhang Y. (2013). Atypical chemokine receptors predict lymph node metastasis and prognosis in patients with cervical squamous cell cancer. Gyn. Oncol..

[27] Zeng X.H., Ou Z.L., Yu K.D., Feng L.Y., Yin W.J., Li J., Shen Z.Z., Shao Z.M. (2011). Coexpression of atypical chemokine binders (ACBs) in breast cancer predicts better outcomes. Breast Cancer Res. Treat..

[28] Zhu Z., Sun Z., Wang Z., Guo P., Zheng X., Xu H. (2013). Prognostic impact of atypical chemokine receptor expression in patients with gastric cancer. J. Surg. Res..

[29] Wu F.Y., Ou Z.L., Feng L.Y., Luo J.M., Wang L.P., Shen Z.Z., Shao Z.M. (2008). Chemokine decoy receptor d6 plays a negative role in human breast cancer. Mol. Cancer Res..

[30] Shi J.Y., Yang L.X., Wang Z.C., Wang L.Y., Zhou J., Wang X.Y., Shi G.M., Ding Z.B., Ke A.W., Dai Z. (2015). CC chemokine receptor-like 1 functions as a tumour suppressor by impairing CCR7-related chemotaxis in hepatocellular carcinoma. J. Pathol..

[31] Ju Y., Sun C., Wang X. (2019). Loss of atypical chemokine receptor 4 facilitates C-C motif chemokine ligand 21-mediated tumor growth and invasion in nasopharyngeal carcinoma. Exp. Ther. Med..

[32] Bednarz-Misa I., Diakowska D., Krzystek-Korpacka M. (2019). Local and systemic IL-7 concentration in gastrointestinal-tract cancers. Medicina.

[33] Bednarz-Misa I., Diakowska D., Szczuka I., Fortuna P., Kubiak A., Rosińczuk J., Krzystek-Korpacka M. (2020). Interleukins 4 and 13 and Their Receptors Are Differently Expressed in Gastrointestinal Tract Cancers, Depending on the Anatomical Site and Disease Advancement, and Improve Colon Cancer Cell Viability and Motility. Cancers.

[34] Bednarz-Misa I., Fortuna P., Diakowska D., Jamrozik N., Krzystek-Korpacka M. (2020). Distinct Local and Systemic Molecular Signatures in the Esophageal and Gastric Cancers: Possible Therapy Targets and Biomarkers for Gastric Cancer. Int. J. Mol. Sci..

[35] Mao L., Clark D. (2015). Molecular margin of surgical resections—Where do we go from here?. Cancer.

[36] Dakubo G.D., Jakupciak J.P., Birch-Machin M.A., Parr R.L. (2007). Clinical implications and utility of field cancerization. Cancer Cell Int..

[37] Patel A., Tripathi G., Gopalakrishnan K., Williams N., Arasaradnam R.P. (2015). Field cancerisation in colorectal cancer: A new frontier or pastures past?. World J. Gastroenterol..

[38] Feng L.Y., Ou Z.L., Wu F.Y., Shen Z.Z., Shao Z.M. (2009). Involvement of a novel chemokine decoy receptor CCX-CKR in breast cancer growth, metastasis and patient survival. Clin. Cancer Res..

[39] Greystoke A., Mullamitha S.A. (2012). How many diseases are colorectal cancer?. Gastroenterol. Res. Pract..

[40] Yamauchi M., Lochhead P., Morikawa T., Huttenhower C., Chan A.T., Giovannucci E., Fuchs C., Ogino S. (2012). Colorectal cancer: A tale of two sides or a continuum?. Gut.

[41] Simons C.C., Hughes L.A., Smits K.M., Khalid-de Bakker C.A., de Bruïne A.P., Carvalho B., Meijer G.A., Schouten L.J., van den Brandt P.A., Weijenberg M.P. (2013). A novel classification of colorectal tumors based on microsatellite instability, the CpG island methylator phenotype and chromosomal instability: Implications for prognosis. Ann. Oncol..

[42] Krzystek-Korpacka M., Diakowska D., Grabowski K., Gamian A. (2012). Tumor location determines midkine level and its association with the disease progression in colorectal cancer patients: A pilot study. Int. J. Colorectal. Dis..

[43] Krzystek-Korpacka M., Diakowska D., Kapturkiewicz B., Bębenek M., Gamian A. (2013). Profiles of circulating inflammatory cytokines in colorectal cancer (CRC), high cancer risk conditions, and health are distinct. Possible implications for CRC screening and surveillance. Cancer Lett..

[44] Krzystek-Korpacka M., Zawadzki M., Kapturkiewicz B., Lewandowska P., Bednarz-Misa I., Gorska S., Witkiewicz W., Gamian A. (2018). Subsite heterogeneity in the profiles of circulating cytokines in colorectal cancer. Cytokine.

[45] Huang C.W., Tsai H.L., Huang M.Y., Huang C.M., Yeh Y.S., Ma C.J., Wang J.Y. (2015). Different clinicopathologic features and favorable outcomes of patients with stage III left-sided colon cancer. World J. Surg. Oncol..

[46] Catalano V., Loupakis F., Graziano F., Torresi U., Bisonni R., Mari D., Fornaro L., Baldelli A.M., Giordani P., Rossi D. (2009). Mucinous histology predicts for poor response rate and overall survival of patients with colorectal cancer and treated with first-line oxaliplatin- and/or irinotecan-based chemotherapy. Br. J. Cancer..

[47] Phipps A.I., Chan A.T., Ogino S. (2013). Anatomic subsite of primary colorectal cancer and subsequent risk and distribution of second cancers. Cancer.

[48] Savino B., Caronni N., Anselmo A., Pasqualini F., Borroni E.M., Basso G., Celesti G., Laghi L., Tourlaki A., Boneschi V. (2014). ERK-Dependent downregulation of the atypical chemokine receptor D6 drives tumor aggressiveness in Kaposi sarcoma. Cancer Immunol. Res..

