# Supplementary material for: Biphasic Expression of Atypical Chemokine Receptor (ACKR) 2 and ACKR4 in Colorectal Neoplasms in Association with Histopathological Findings
Source: Biomolecules. 2020 Dec 23;11(1):8. doi: 10.3390/biom11010008 (PMC7824260; doi:10.3390/biom11010008)
Supplement: Supplementary file 1 [file biomolecules-11-00008-s001.pdf]

Supplementary Materials

for Paulina Lewandowska et al. “Biphasic Expression of Atypical Chemokine Receptor (ACKR) 2 and ACKR4 in Colorectal Neoplasms in Association with Histopathological Findings”

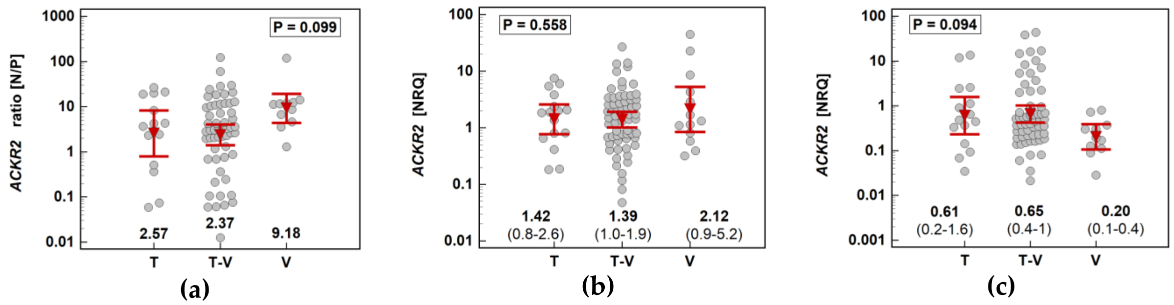

Figure S1. Impact of adenoma histological type on *ACKR2* expression: (**a**) Normal–to-polyp expression ratio [N/P]; (**b**) In normal tissue; (**c**) In adenomas. Data presented as dot-plots with means (95% confidence interval) and analyzed using one-way analysis of variance. T, tubular adenomas; T-V, tubulo-villous adenomas; V, villous adenomas; NRQ, normalized relative quantity.

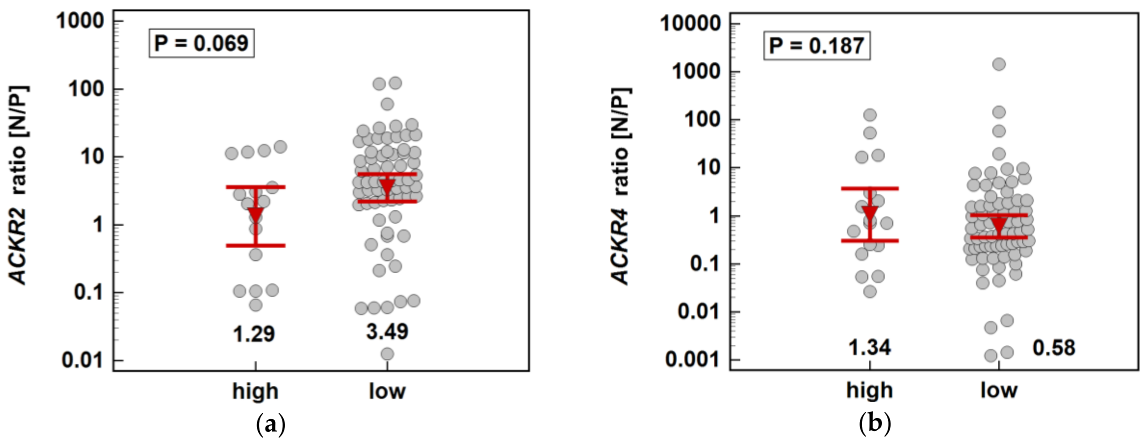

Figure S2. Impact of dysplasia grade on adenoma expression of: (**a**) *ACKR2*; (**b**) *ACKR4*. Data presented as dot-plots and mean normal-to-polyp [N/P] expression ratio and analyzed using t-test for independent samples.

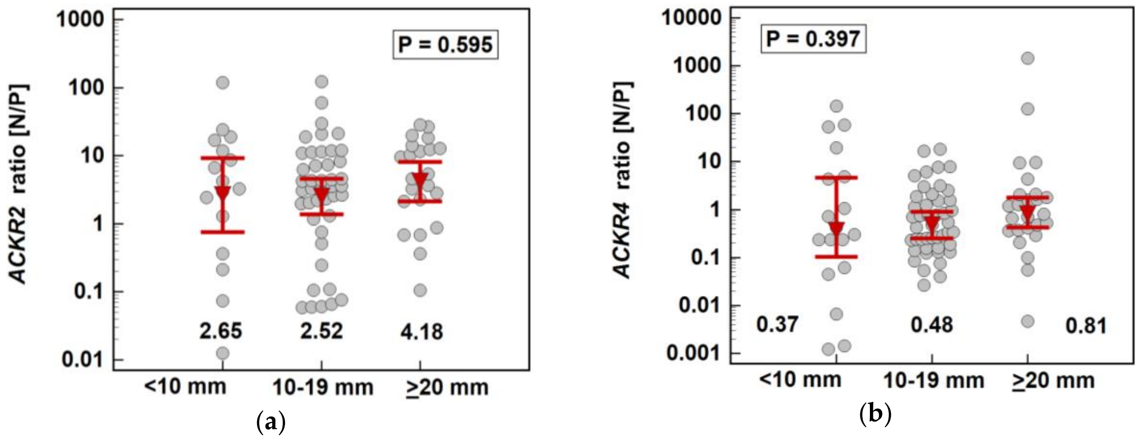

Figure S3. Impact of adenoma size on the expression of: (**a**) *ACKR2*; (**b**) *ACKR4*. Data presented as dot-plots and mean (*ACKR2*) or median (*ACKR4*) normal-to-polyp [N/P] expression ratio and analyzed using one-way analysis of variance or Kruskal-Wallis H test, respectively.

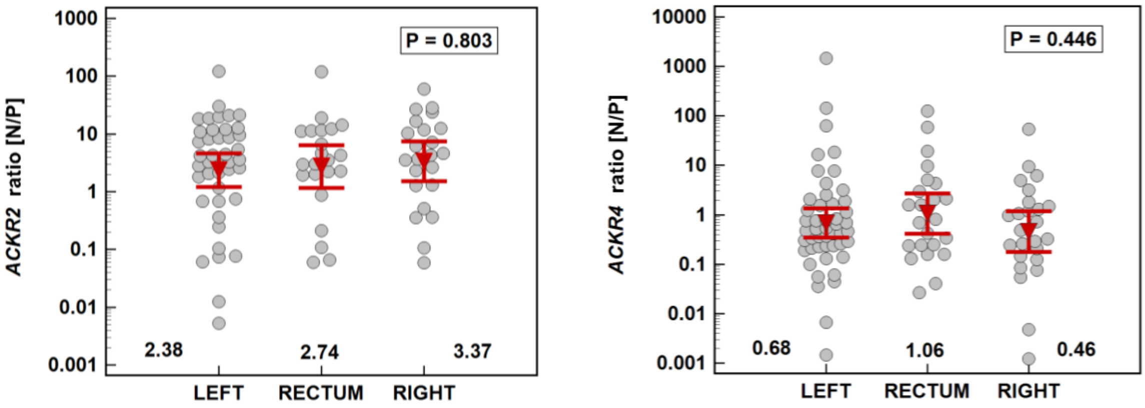

(**a**) (**b**)

Figure S4. Impact of adenoma location on the expression of: (**a**) *ACKR2*; (**b**) *ACKR4*. Data presented as dot-plots and mean normal-to-polyp [N/P] expression ratio and analyzed using one-way analysis of variance.

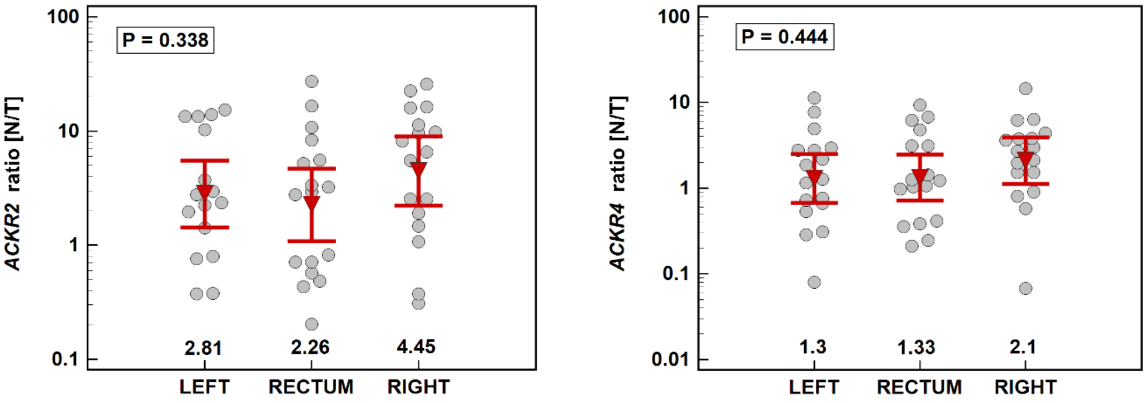

(**a**) (**b**)

Figure S5. Impact of tumor location on the expression of: (**a**) *ACKR2*; (**b**) *ACKR4*. Data presented as dot-plots and mean normal-to-tumor [N/T] expression ratio and analyzed using one-way analysis of variance.

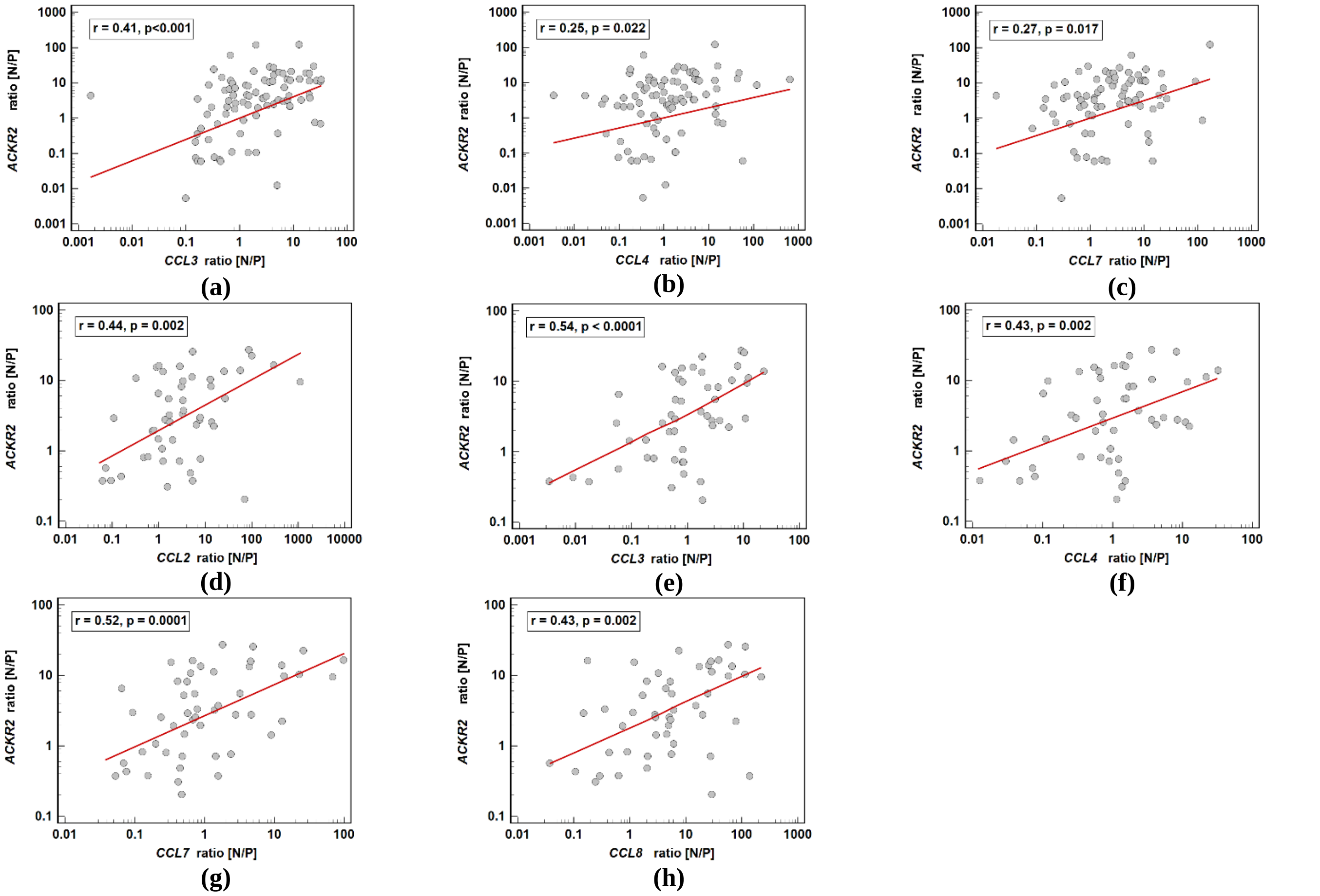

Figure S6. Correlation between *ACKR2* and chemokine expression: (**a**) *CCL3* in polyps; (**b**) *CCL4* in polyps; (**c**) *CCL7* in polyps; (**d**) *CCL2* in adenocarcinomas; (**e**) *CCL3* in adenocarcinomas; (**f**) *CCL4* in adenocarcinomas; (**g**) *CCL7* in adenocarcinomas; (**h**) *CCL8* in adenocarcinomas. Data presented as scatter-plots with a trend line and Pearson correlation coefficient (r). N/T, normal-to-tumor expression ratio.
